# Effects of oil-film layer and surfactant on the siphonal respiration and survivorship in the fourth instar larvae of *Aedes togoi* mosquito in laboratory conditions

**DOI:** 10.1038/s41598-018-23980-5

**Published:** 2018-04-09

**Authors:** Sang Joon Lee, Jun Ho Kim, Seung Chul Lee

**Affiliations:** Department of Mechanical Engineering, Pohang University of Science and Technology, Phoang, Gyeongbuk, Republic of Korea

## Abstract

Mosquitoes transmit various diseases; thus, controlling them is necessary to prevent mosquito-borne infections. Unlike flying adult mosquitoes, those in the immature stages are easy to control because of being restricted to their habitats found in an aquatic environment. In this study, we aimed to evaluate of respiration and survivorship in the larvae of *Aedes togoi*. The mechanism of actions of the oil-film layer and the surfactant as well as their effects on the siphonal respiration of submerged *Aedes togoi* larvae were analyzed by checking the survival time of mosquito larvae against oil-film layer and surfactant, and conducting experiments using a siphon-model. Compared with an impermeable membrane used for reference (762.4 min; average time in all cases), the survival time of mosquito larvae was 5% longer for the oil-film layer (808.1 min) and 40% longer for the surfactant (1086.9 min). The surface of the siphon was changed from hydrophobic to hydrophilic by addition of a surfactant. In addition, the surface tension and wettability have a significant influence on the opening and closing of siphon. This study would be helpful for understanding the basic mechanism of physical control measures for disturbing the siphonal respiration of mosquito larvae in a way of dissolved oxygen and surface tension. The present results would guide the establishment of effective control measures for mosquitoes.

## Introduction

Mosquitoes transmit various vector-borne diseases such as dengue fever, Japanese encephalitis, malaria, and Zika virus^1^; hence, controlling them is crucial to prevent mosquito-borne infections. *Aedes togoi*, which is a target mosquito species of this study, is mainly involved in the transmission of a wide species of filariae, yellow fever, and Japanese encephalitis in Southeast Asia, the Pacific coast of Canada and USA^2,3^. Anthropogenic dispersal may have caused in the wide spreading of the aedine species *Aedes togoi*, which is breeding in artificial containers as well as coastal rock pools, across climatic zones^2^. In really, the *Aedes togoi* is widely distributed from subarctic East Asia to subtropical. In addition, the occurrence of *Aedes togoi* has also been reported from the Pacific coast of Canada and the USA^2,3^. A conventional way to prevent vector-borne diseases is to target adult female mosquitoes with insecticides to reduce the lifetime of vectors and the chance to feed on human blood^4^. However, flying adult mosquitoes are difficult to catch and they exhibit strong resistance to insecticides^4–6^. On the other hand, given that mosquitoes in the immature stages (eggs, larvae, and pupae) are restricted to small-scale aquatic habitats, avoiding the control measures is difficult for them^4–6^. Therefore, one alternative to prevent mosquito-borne diseases is to target mosquitoes at the immature stages. Killeen *et al*. reported a documented example of larval control for sustained and successful malaria prevention in sub-Saharan Africa and this larval control endeavor predates the advent of dichlorodiphenyltrichloroethane (DDT)^4^.

The control measures for mosquitoes involve chemical control, biological control, environmental management, genetic control, and physical control^7^. Among the control measures, several methods have been controversial because of ecosystem disturbance and the tolerance development of mosquitoes against the given control methods^6–8^. However, mosquitoes have not acquired tolerance against physical control methods^7^. Oil, surface film, and polystyrene beads have been introduced to disturb the respiration of mosquito larvae and pupae submerged in water^6,7,9–12^.

Certain species of mosquito larvae breathe underwater by piercing their air tube called a siphon^12^. *Aedes* mosquito larva was reported to get air through its siphon with floating the apical part of the siphon for breathing^13^. One way to control them is to physically interrupt respiration by disturbing their contact with air above the water surface^13^. However, previous study on the use of oil to kill mosquito larvae only focused on the toxicity of oil^6^. Effective methods for disturbing the respiration of mosquito larvae were also not elucidated. Therefore, a detailed understanding of the physical control methods to disturb mosquito larvae respiration will be helpful in choosing an effective control method.

In the present study, we investigated effective methods for disturbing the respiration of mosquito larvae. In addition, we analyzed the force balance between Laplace pressure as well as hydrostatic pressure that causes tracheal flooding mathematically and verified experimentally. The present results would be helpful in understanding the physical control measures for mosquitoes and the design of an effective physical control device.

## Result

### Morphological characteristics of the lobes of the siphon

**Figure 1:**
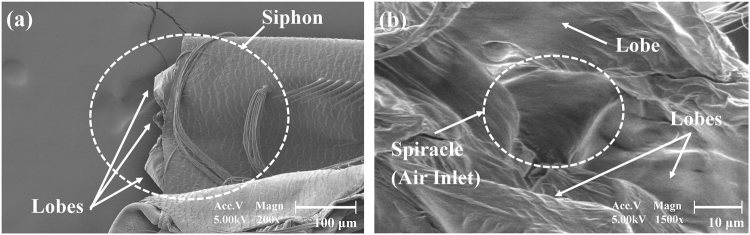
Typical SEM images of the siphon and lobes of *Ae*. *togoi* mosquito larva. SEM image of the siphon part (600X magnification) (**a**) and the spiracle part (1500X magnification) (**b**).

Figure 1 shows the scanning electron microscopy (SEM) images of the siphon of an *Aedes togoi* mosquito larva. The tip of the siphon consisted of lobes and a spiracle, which is the air inlet. The spiracle was surrounded by three big lobes, two posterior perispiracular lobes, and an anterior perispiracular lobe. The lobes were closed to form a hollow cone-shaped air cavity to protect the mosquito larva by preventing water from penetrating the spiracle as the base of the hollow cone.

### Survival times of mosquito larvae in the water

**Figure 2:**
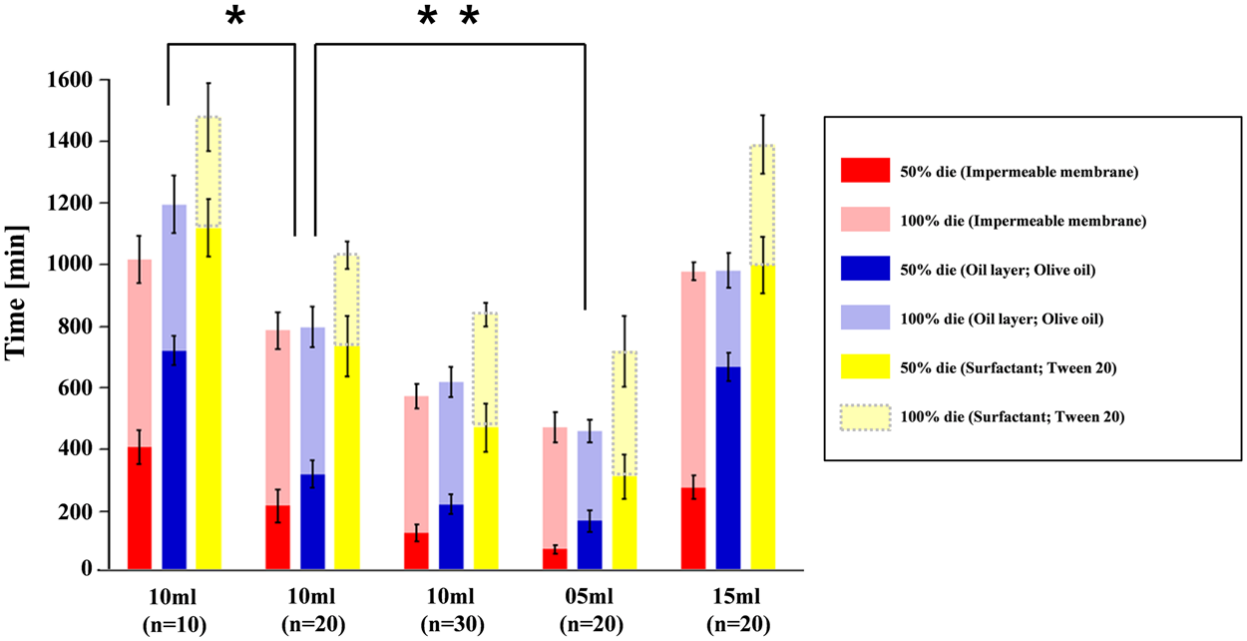
Comparison of the survival times for three treatments. [1] impermeable membrane (red bar), [2] oil layer (blue bar), [3] surfactant (yellow bar). The error bar represents the standard deviation (*P < 0.05, **P < 0.05, n = 5).

**Figure 3:**
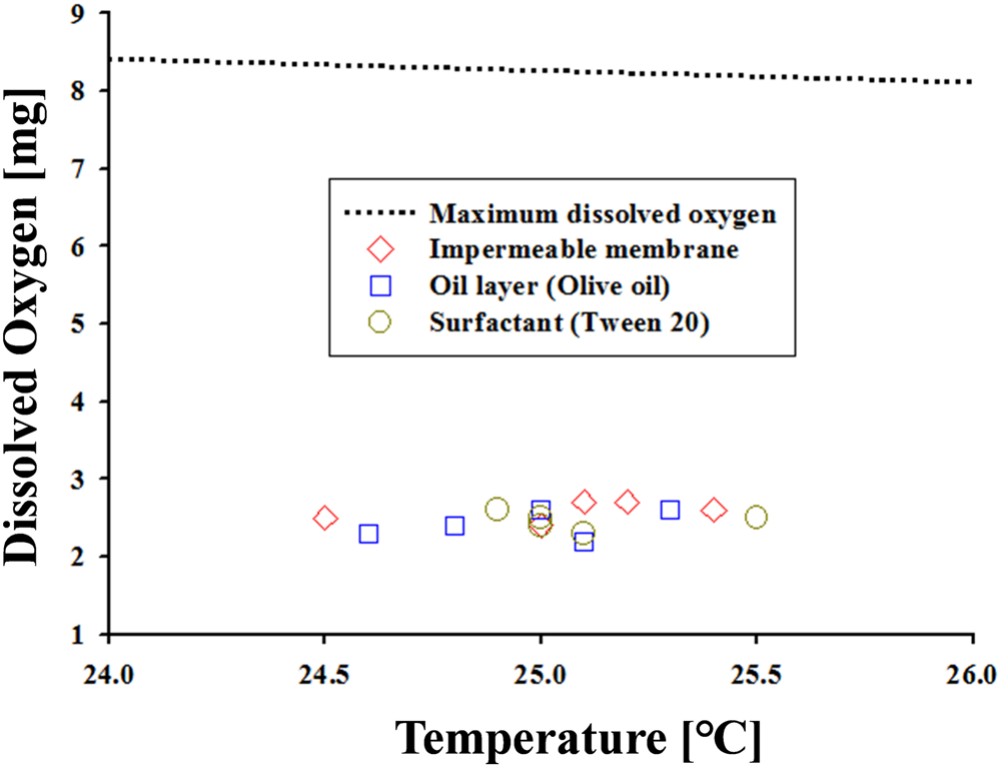
Variations of dissolved oxygen measured after all mosquito larvae are dead. The dashed line indicates the theoretical maximum dissolved oxygen per 1$\ell$.

The survival times of mosquito larvae in an aquatic environment were compared in three different treatments. In 10 m$\ell$ distilled water saturated with oxygen, the averaged times of dying for 50% of the 10 larvae were 408.6 ± 54.5 min, 718.2 ± 47.5 min, and 1114.8 ± 93.9 min, with the use of impermeable membrane, oil-film layer, as well as surfactant, respectively. The respective elapsed times of dying for all the larvae were 1011.6 ± 76.1 min, 1191.0 ± 93.9 min, and 1471.8 ± 109.3 min, respectively (Fig. 2, Table S1). Based on these results, the survival time of mosquito larvae was about 5% longer for the oil-film layer and about 40% longer for the surfactant compared with an impermeable membrane used for reference. Experiments for the control group, which did not take any treatment, were conducted for 3 days, and the natural mortality rate was observed to be less 2%. For the impermeable membrane, which does not allow the generation of dissolved oxygen and the respiration of mosquito larvae, survival time was the shortest, excluding one case (20 larvae in 5m$\ell$distilled water). In addition, the survival time decreased as the volume of water decreases or the population of mosquito larvae increases (*P < 0.05, **P < 0.05). This result supports that the use of oil-film layer and surfactant is effective in controlling mosquito larvae. The content of dissolved oxygen in the water was also measured after conducting all the experiments. The dissolved oxygen was ranged from 27.58 to 32.73% (29.79 ± 1.77%) (Fig. 3). However, these results were obtained by considering dissolved oxygen only, excluding the other factor under laboratory conditions.

### Gating mechanism of lobes

**Figure 4:**
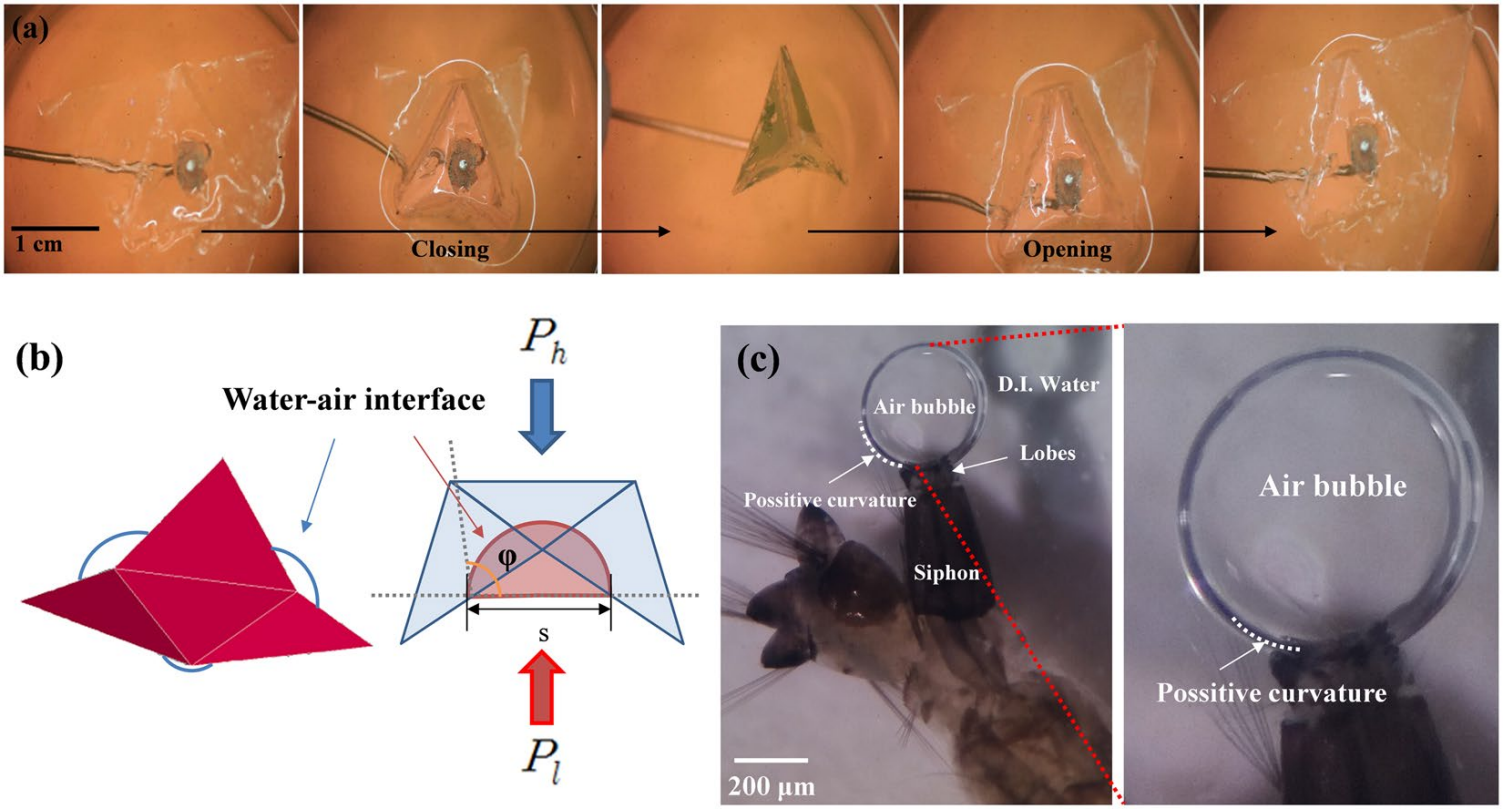
The opening and closing mechanism of the siphon model. (**a**) Five consecutive images of the siphon model moving near the water-air interface. (**b**) Schematic illustration of the force balance between hydrostatic pressure (*P*_*h*_) and Laplace pressure (*P*_*l*_) . (**c**) Wettability of the siphon, especially the lobes part, after dying with surfactant. The white dashed line represents a positive curvature of gas bubble released from the spiracle.

To simulate the operation of a real siphon opened and closed by three large lobes (and two small lobes) with a hydrophobic surface, we fabricated a siphon model using a spin coating method and observed its dynamic behavior in a water-air interface (Fig. 4a, Supp. S1). When the siphon opened and closed, the hydrostatic pressure (*P*_*h*_) of water and the Laplace pressure (*P*_*l*_) are balanced in a competitive manner. The hydrostatic pressure promotes water entrance into the cone-shaped cavity in the siphon through the haps among the three lobes. On the other hand, the Laplace pressure is applied at the boundary between the gas and liquid regions to prevent the intrusion of water into the siphon against hydrostatic pressure (Fig. 4b). When the maximum hydrostatic pressure is lower than the minimum Laplace pressure, tracheal flooding does not occur. This situation can be expressed as follow;

1 $$\begin{array}{c}{P}_{h,maximum}\le {P}_{l,minimum}\\ {\rm{\rho }}{\rm{g}}\sqrt{\frac{2}{3}}{\rm{a}}\,\,\,\,\le \frac{2\sigma sin\phi }{\frac{\sqrt{2}}{2}a}\\ \quad \quad {\rm{a}}\approx {10}^{-2}{\rm{m}}\end{array}$$

where ρ is density of water, g is the gravitational acceleration, *σ* is the surface tension, a is the side length of the triangle of siphon model, and *φ* is the contact angle (ρ = 996.9 kg/*m*^3^, g = 9.81 m/*s*^2^, *σ* = 0.072 N/m at 25 °C).

The side length of the siphon model’s triangle was 1.5–2.5 cm. Consequently, the larger the side length of the triangle, the less reproducible the experiments. This finding confirmed that the aforementioned mathematical analysis is valid.

We tried to apply the above mathematical modeling to control mosquito larvae using a surfactant. In this case, the following relationship should be satisfied to induce the tracheal flooding of mosquito larvae by using a surfactant.

2 $$\begin{array}{c}{P}_{h} > {P}_{l}\\ \sigma \approx {10}^{(-4)}\,{\rm{N}}/{\rm{m}}\end{array}$$

Generally, the addition of surfactant decreases the surface tension of water. However, this activity does not change the order of surface tension (*σ* = 0.04 N/m at 25 °C at the critical micelle concentration (CMC) of Tween 20). Therefore, in order to cause tracheal flooding of mosquito by using surfactants, the surface tension and other parameters should be changed. When the surfactants were added, the surface tension of water and wettability of the siphon including the lobes of mosquito larvae changed (Fig. 4c). The air bubble positioned on the submerged lobes had a positive curvature (marked on the white dotted line in Fig. 4c). This scenario implied that the wettability of the siphon, including the lobes, changed from hydrophobicity^14^ to hydrophilicity with the addition of the surfactant.

### Dynamic behavior of lobes at the oil-water interface and lethal effects of oil-film layer

**Figure 5:**
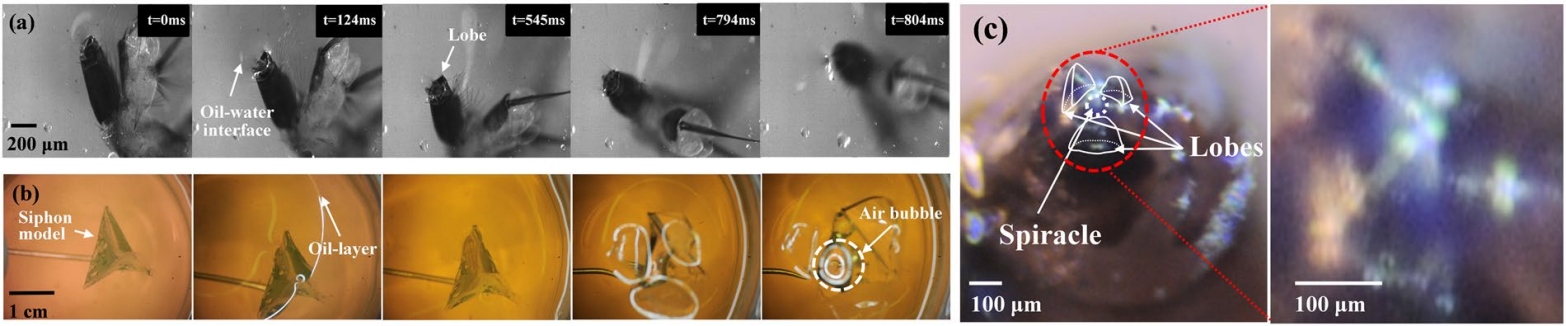
Dynamic behavior of the siphon of a mosquito larva at the oil-water interface under the application of oil-film. (**a**) Series of magnified images showing dynamic motion of the siphon of *Ae. togoi* mosquito larva at the oil-water interface. (**b**) Consecutive images showing dynamic motion of the siphon model in the region near the oil-water interface. (**c**) Occurrence of tracheal flooding caused by oil-film layer containing blue dye.

*In vivo* and model experiments were carried out to investigate the mosquito killing ability of an oil-film layer. Initially, we conducted an *in vivo* experiment using living mosquito larvae (Fig. 5a, Supp. S2). Figure 5a shows a series of magnified images consecutively captured by a high-speed camera of a mosquito larva’s siphon opened at the oil-water interface. When the siphon model was placed in the water with closed lobes, we formed an oil-film layer on the water surface (Fig. 5b, Supp. S3). Subsequently, we slowly raised the siphon model toward the water surface. In Fig. 5b, the siphon model starts to open at the oil-water interface, and a gas bubble was produced by the air contained in the siphon’s air cavity. The end part of the siphon was opened when the latter reached the oil-water interface. Thereafter, we carried out another experiment using diluted oil in blue dye to directly check whether the tracheal flooding would occur. Using the same experimental procedures employed for measuring the survival time of mosquito larvae, we examined mosquito larvae in the water with a top layer of oil-film diluted in blue dye. When the mosquito larva sample died, we opened the lobes as well as observed the presence of blue-dyed oil on its inner surface and spiracle (Fig. 5c).

## Discussion

Among all the other control methods, only the physical methods disallow mosquito larvae to acquire tolerance^15^. Although they directly disturb the siphonal respiration, the detailed mechanism of breathing cut-off for mosquito larvae was not elucidated. Accordingly, we focused on the use of oil-film layer and surfactant as the physical mosquito control methods.

A previous study asserted that surface tension and wettability significantly contribute to the opening and closing of the larvae’s siphon for certain mosquito species^13^. In the present study, the use of oil-film layer and surfactant (Tween 20) was confirmed to be effective in killing mosquito larvae. The use of a surfactant would affect the wettability of the siphon, including the lobes of mosquito larvae. The wettability of the siphon was changed from hydrophobic to hydrophilic with the addition of the surfactant.

The result of dissolved oxygen concentration (29.79 ± 1.77%), after all the mosquito larvae died, is well matched with the previous study in which the critical dissolved oxygen concentration was approximately 30%^14^. The cutaneous respiration seems to adequately compensate for the absence of siphonal respiration. These results prove that mosquito larvae fail to do siphonal respiration with the application of an oil-film layer or a surfactant. Consequently, mosquito larvae mainly depend on the dissolved oxygen in water. Eventually, mosquito larvae die when the concentration of dissolved oxygen reaches a critical level (roughly 30%). However, these results were obtained by considering only dissolved oxygen with treating oxygen in the air to prevent to dissolving in water under laboratory conditions. Therefore, if other factors are involved, different results could be derived. For example, if toxic essential oils such as camphor (*Cinnamomum camphora*), lemon (*Citrus limon*), and dill (*Anethum graveolens*) are used instead of olive oil used in this study, the correlation between DO and mortality rate would become meaningless^6^. In addition, the effects of other factors including temperature, larval instar, food, and carcass should be considered at further studies in the future.

Among the oil-film layer, surfactant, and impermeable membrane, which block the production of dissolved oxygen as well as siphonal respiration, the impermeable membrane has the shortest survival time, excluding one case. When oil-film layer and surfactant were used, the oxygen component in the air is not completely blocked from being dissolved in water. However, when the cases of oil-film layer and surfactant were compared with each other, the survival time in the oil-film layer method is shorter than that in the surfactant method. These results corroborate that the effect of preventing dissolved oxygen generation to a certain extent can be obtained when the oil-film layer is applied. In addition, contrary to the previous studies that focused on the toxicity and use of oils^6^, the proposed oil-film layer is effective for preventing their siphonal respiration.

Compared with the use of oil-film layer, the use of surfactant is inferior in killing ability. However, Tween 20 (Polysorbate 20), commonly threated as a group of Polysorbate, is regarded safe^16^ because no difference in the disposition or adverse effects is observed among its group^17^. In addition, only a small amount of 71.2 g of Tween 20 (CMC; 0.058 mM at 25 °C, molecular weight; 1227.54 g/mol)^18^ is required to reach the CMC of 1000 $\ell$ of water. This scenario denotes that a small amount of Tween 20 is enough to be an effective control measure of mosquito larvae.

Considering the application of control technology to the field, a large amount of oil is required to form a layer of oil-water interface given that the oil-film method has a better killing ability compared with the surfactant method. On the other hands, a small amount of surfactant is used to reach the CMC of the water. Therefore, to control mosquito larvae in a small aquatic area of mosquito habitats, we should employ the oil-film method for short-term treatment. Otherwise, the use of surfactants appears to be cost-effective and easy to use in large mosquito habitat areas. However, according to previous study^19,20^, some mosquitoes at immature stages are found in a paddle, bamboo holes, and animal footprints etc. In these cases, to apply the present methods in this study may have a limitation in their applications. In this study, surface tension and dissolved oxygen were only considered for fourth instar larvae of *Aedes togoi* under constant temperature and relative humidity condition. Thus, this study has such technical limitations. In future studies, other factors such as mosquito species diversity, larval instar, and the real environment conditions where mosquitoes live should be additionally considered.

In the study, the mosquito killing ability of surfactant and oil-film layer was demonstrated, and the principle of the present control methods was elucidated. The present study on the proposed physical control’s mechanism would be helpful in guiding the establishment of effective mosquito control measures.

## Method

### Mosquito larvae rearing and sample preparation

Korea Center for Disease Control and Prevention provided the mosquito eggs, and approximately 150 to 200 mosquito larvae (*Aedes togoi*) were reared in a 2$\ell$ plastic container, which were fed with a slurry of baker’s yeast and ground fish food. Mosquito larvae were reared under the conditions of 27 °C, 80% relative humidity and a 16 h: 8 h light/dark cycle. The 4^th^ instar mosquito larvae were used in the experiment.

### Measuring the survival time of mosquito larvae

Mosquito larvae were placed in a centrifuge tube (15mlL) containing water saturated with oxygen. The water used in the experiment was de-ionized water (type III), the temperature was 25 ± 0.2 °C, and initial amount of dissolved oxygen was 7.7 ± 0.1 mg per 1lL. Before each experiment, *Aedes* mosquito larvae were fed with a slurry of baker’s yeast and ground fish food (more than 0.2 mg per larva), which is more than the standard amount (0.09 mg per larva)^21^. To investigate the effects of oil-film layer (olive oil) and surfactant (Tween 20), we captured three images every 3 min to see whether the mosquito larvae die. We classified each mosquito larva as dead when the latter does not show movement for 15 minutes (through 45 consecutive pictures). The thickness of the tested oil-film layer was 5 mm, and the surfactant Tween 20 was added to water until it reached the critical micelle concentration (CMC; 0.058 mM at 25 °C)^20^. The experimental results obtained from oil-film layer and surfactant were compared with those from an impermeable membrane, which did not allow the generation of dissolved oxygen and respiration of mosquito larvae. All the experiments were conducted in a constant temperature and humidity chamber (temperature: 25 ± 0.26 °C, relative humidity: 50 ± 0.97%). At the end of each experiment, the dissolved oxygen content of the water was measured with dissolved oxygen meter (PDO-520, Lutron, USA). All data are expressed as the mean value ± standard deviation represented by an error bar (n = 5).

### Scanning electron microscopy

A scanning electron microscopy (SEM) was used to measure the thickness of the siphon model and observe the morphological configuration of the mosquito larvae samples. Larval samples were prepared by dehydration using a series of ethanol solution (70%, 80%, 90%, and 99%, overnight each) and chemical drying using HMDS (hexamethyldisilazane). The test samples were then Ag-coated using a coater (Quorum Technology, SC7640 mode, East Sussex, United Kingdom) and examined by a field emission SEM (XL30S FEG, Philips Electron Optics B.V., the Netherlands) connected to an EDXS system at an acceleration voltage of 5.0 kV.

### Fabrication of siphon model

A siphon model was made of low**-**density polyethylene (LDPE) coated with PDMS by spin coating method to control the wettability of the model surface. The PDMS base was mixed with a curing agent at a weight ratio of 10:1. The protocol of the spin coating method is as follow;

**Step 1:** 500 rpm at 100 rpm/s (acceleration) for 20 seconds.

**Step 2:** 2400 rpm at 100 rpm/s for 20 seconds.

**Step 3:** Hold for 10 minutes.

**Step 4:** Heat in a 40 °C oven for 6 hours for annealing.

The thickness of the PDMS coated on LDPE of 19.81 ± 0.55 μm is 18.87 ± 1.03 μm. The wettability of the siphon model was checked by measuring its contact angle with water droplet, which was measured at 113.46 ± 3.69 degree. The side length of the triangle of the siphon model tested in this study is 1.5–2.5 cm.

### Dynamic motion of the siphon at the water-oil interface

Living larvae were placed in a microtube containing distilled water saturated with air at the given temperature. The microtube was located on the stage of a microscope (Eclipse 80i, Nikon, Japan) attached to a high-speed camera (Fastcam Sa1.1, Photron, USA). The microscope focused on the water-oil (olive oil) interface to see dynamic behaviors of the mosquito larvae siphon, which were consecutively recorded in top view at a frame rate of 1000 frames/s. The starting point of the floating process (t = 0 ms) was defined, when the water-oil interface was distorted by the contact of the siphon lobes. The field of view is 1024 μm × 1024 μm, and the captured high-speed images’ contrast was enhanced using digital image-processing techniques.

### Statistical analysis

Experimental data about the survival times of mosquito larvae were statistically analyzed using t-test (statistical method used when population variance and standard deviation are unknown) with 95% confidence interval in SPSS (IBM, Chicago, IL, USA). The p-value acquired by t-test for all cases of this study is less than 0.05. This indicates that the acquired experimental data hold meaningful information with 95% confidence.

## Electronic supplementary material

Table S1

Movie S1

Movie S2

Movie S3
