# Supplementary material for: Effects of oil-film layer and surfactant on the siphonal respiration and survivorship in the fourth instar larvae of Aedes togoi mosquito in laboratory conditions
Source: Sci Rep. 2018 Apr 9;8:5694. doi: 10.1038/s41598-018-23980-5 (PMC5890275; doi:10.1038/s41598-018-23980-5)
Supplement: Supplementary file 1 — Table S1 [file 41598_2018_23980_MOESM1_ESM.docx]

**Effects of oil-film layer and surfactant on the siphonal respiration and survivorship in the fourth instar larvae of *Aedes togoi* mosquito in laboratory conditions**

Sang Joon Lee^1,†^, Jun Ho Kim^1^, and Seung Chul Lee^1^

^1^Department of Mechanical Engineering, Pohang University of Science and Technology, Phoang, Gyeongbuk, Republic of Korea.

E-mail addresses

Sang Joon Lee: [sjlee@postech.ac.kr](mailto:sjlee@postech.ac.kr)

Jun Ho Kim: [junho8903@postech.ac.kr](mailto:junho8903@postech.ac.kr)

Seung Chul Lee: leesch00@postech.ac.kr

**^†^**Corresponding author: **Prof. Sang Joon Lee**

Department of Mechanical Engineering, Pohang University of Science and Technology, San 31, Hyojadong, Namgu, Pohang, Gyungbook, 790-784, Republic of Korea

Tel.: +82-54-279-2169, Fax: +82-54-279-3199, E-mail: sjlee@postech.ac.kr

|  |  | **Survival time [min]** | | | | | | | | |
| --- | --- | --- | --- | --- | --- | --- | --- | --- | --- | --- |
| **Treatment** |  | **10ml (n=10)** |  | **10ml (n=20)** |  | **10ml (n=30)** |  | **5ml (n=20)** |  | **15ml (n=20)** |
| **Impermeable membrane** |  | 1011.6$\pm$76.07 |  | 783.6$\pm$59.92 |  | 571.8$\pm$40.19 |  | 471.0$\pm$49.66 |  | 973.8$\pm$29.00 |
|  |  |  |  |  |  |  |  |  |  |  |
| **Oil-layer**  **(Olive oil)** |  | 1191.0$\pm$93.85 |  | 795.0$\pm$65.04 |  | 617.4$\pm$50.18 |  | 459.0$\pm$36.45 |  | 978.0$\pm$56.12 |
|  |  |  |  |  |  |  |  |  |  |  |
| **Surfactant (Tween 20)** |  | 1471.8$\pm$109.27 |  | 1026.6$\pm$44.8 |  | 835.8$\pm$38.73 |  | 717.0 $\pm$114.27 |  | 1383.0$\pm$94.30 |

**Table S1.** Comparison of survival times of mosquito larvae (*Aedes togoi*) for three treatments**;** Mean $\pm s$tandard deviation (n=5)
